# A Flexible Traffic Signal Coordinated Control Approach and System on Complicated Transportation Control Infrastructure

**DOI:** 10.3390/s23135796

**Published:** 2023-06-21

**Authors:** Songhang Chen, Chunlin Shang, Fenghua Zhu

**Affiliations:** 1 Fujian Institute of Research on the Structure of Matter, Chinese Academy of Sciences, Fuzhou 350002, China; songhang.chen@fjirsm.ac.cn; 2 The State Key Laboratory of Multimodal Artificial Intelligence Systems, Institute of Automation, Chinese Academy of Sciences, Beijing 100190, China; itsshang@ldu.edu.cn

**Keywords:** regional traffic signal control, transportation control infrastructure, flexible, universal adaptability

## Abstract

The transportation control infrastructure serves as the foundation for regional traffic signal control. However, in practice, this infrastructure is often imperfect and complex, characterized by factors such as heterogeneity and uncertainty, which pose significant challenges to existing methods and systems. Therefore, this paper proposes a novel approach to coordinated traffic signal control that emphasizes flexibility. To achieve this flexibility, we combine the flexible model of complex networks with robust fuzzy control methods. This approach enables us to overcome the complexity of the transportation control infrastructure and ensure efficient management of traffic signals. Additionally, to ensure long-term operational ease, we develop a regional traffic signal control system using steam computing technology, which provides high scalability and compatibility. Finally, computational experiments are performed to validate adaptability and performance of our proposed approach.

## 1. Introduction

The regional traffic signal control (RTSC) [1,2,3] heavily relies on transportation control infrastructure, which consists of thousands of devices such as traffic detectors, signal controllers, lights, and communication networks. Consequently, RTSC frequently encounters two aspects of complexity:

1. Engineering complexity **(static)**: The transportation control infrastructure, which is geographically dispersed and built over time through cooperation between multiple participants, requires a new RTSC system to integrate traffic signal controllers and detectors from various manufacturers. Furthermore, some areas may have a high density of devices, while others may have sparse coverage. These heterogeneous situations are very common during urbanization and necessitate high compatibility for RTSC systems;
2. Application complexity **(dynamic)**: The operation of the transportation control infrastructure is heavily influenced by environmental factors such as adverse weather, power outages, and traffic accidents. Any hardware or communication failures can affect RTSC operations. Furthermore, some junctions may operate in higher priority modes (e.g., green wave control, manual control) and be out of coordinated control at times. These uncertain situations are unavoidable during long-term operation and require RTSC systems with high flexibility.

The two types of complexity mentioned are significant challenges that must be addressed when deploying RTSC in practice. Otherwise, the RTSC system may struggle to operate effectively and may even be abandoned after a period of use. The urban transportation system is an open and complex giant system [4]. As the city grows, the transportation control infrastructure expands and the number and the frequency of complicated situations increase faster, posing a significant challenge to the existing RTSC methods and systems. However, most recent studies, such as those by Liu [5] and Wang [6], remain limited to the complexity of traffic flow, and there is a lack of studies taking the complexity of the transportation control infrastructure into account from a broader perspective. In the popular fields of V2V (vehicle-to-vehicle) and V2I (vehicle-to-infrastructure) in recent years, scholars have paid attention to complicated infrastructure, such as by studying the impact of the inevitable quality of service (QoS) issues caused by hardware or communication failures on data offloading [7,8] and traffic admission [9].

Therefore, this paper proposes a novel regional traffic signal coordinated control approach and system that can not only optimize the traffic signal but also adapt to the complicated transportation control infrastructure. The contributions of this paper are listed below:

1. A novel flexible regional traffic signal coordinated control method is proposed, focusing on adaptability to the complexity of the actual transportation control infrastructure;
2. An easy-to-maintain RTSC system is created using the steam computing technology, with a focus on the scalability and compatibility of RTSC systems required for actual traffic management;
3. A triple-random principle is proposed to design the testing experiments on simulation software. Both the performance and the adaptability of the proposed approach are validated.

The remainder of this paper is organized as follows. Section 2 reviews the existing RTSC systems and related works. Section 3 and Section 4 propose the model and method, respectively, while Section 5 develops the RTSC prototype system. Section 6 includes experiments to test the effectiveness of the method and system. Finally, Section 7 summarizes the work of this paper and looks forward to the future.

## 2. Related Work

As early as 1963, the first RTSC system came into use in Toronto, Canada. Since then, numerous methods and systems of RTSC have been proposed and adopted in practice. Table 1 provides a list of typical systems [10,11,12,13].

Currently, TRANSYT, SCOOT, and SCATS are the three most widely used systems. TRANSYT was an offline regional fixed-time coordinated control system developed by the British Institute of Transport and Roads in 1968 [14]. The latest version uses a genetic algorithm to optimize the cycle, split, phase offset and sequence. In 1979, SCOOT was developed based on TRANSYT and is now an online adaptive regional coordinated control system [15]. SCOOT analyzes the traffic data collected by the detectors and continuously adjusts the split, cycle, and phase offset using a traffic model and asymptotic optimization method of small steps. SCATS is a real-time scheme selective RTSC system developed by the Road Transport Authority of New South Wales, Australia in the late 1970s [16]. Based on the dynamic comprehensive evaluation of traffic volume and saturation, SCATS selects a suitable timing scheme from the pre-calculated scheme library. SCOOT and SCATS have been used in Chinese cities, such as Beijing, Shanghai, and Guangzhou [17].

The above three systems were developed in the last century and have undergone 30+ years of iterative improvement both in theory research and system development. In this period, plenty of researchers have been devoted to this area, and numerous network-based and intelligent RTSC methods have been proposed [18]. Research in this area can be mainly categorized into two categories: proposing new algorithms for signal controllers and designing coordination mechanisms to organize all the controllers.

In the first category, fuzzy logic [19], rough set [20], neural network [21], genetic algorithm [22], particle swarm optimization [23], game theory [24], expert system [25], self-organizing rules [26], dynamic adaptive programming [27], bilevel dynamic programming [28,29], reinforcement learning [30], complex network [31,32], back-pressure [33], and their variants, have been applied to innovating new algorithms for signal controllers. Recently, inspired the achievements of deep learning in other areas, deep reinforcement leaning based algorithm have aroused much more attention and becomes a hot research topic [34,35]. New methods, such as P-type control [36] and hybrid data-driven fuzzy control [37], also demonstrate potential applications in RTSC. In the second category, parallel AI, distributed problem solving (DPS), and multi-agent systems (MAS) are three main mechanisms [38]. Among these mechanisms, MAS is the most popular and incorporates the domain expertise in the system to achieve the optimal solution [39,40,41]. In MAS, the RTSC system is usually organized using a three-layer hierarchical structure consisting of a junction control layer, a zone control layer, and a region control layer. In this way, the whole traffic control problem can be divided into smaller sub-problems which require less domain expertise.

Though many RTSC methods have been explored, most of them are still far away from practical applications. The main reasons for this are twofold: first, the complexity of the actual transportation control infrastructure has not been adequately addressed; second, the high scalability and compatibility required by RTSC systems in actual long-term operation have not been met. Taking these requirements into account will bring many constraints to the RTSC research. For example, research can no longer assume that it can obtain the traffic conditions of all junctions and roads, and all junctions cannot be assumed to accept unified coordinated control at any time. Almost all methods without sufficient flexibility do not work anymore, and this situation prompted us to carry out the work in this paper.

## 3. Model

### 3.1. Phase-Based Control

**Figure 1:**
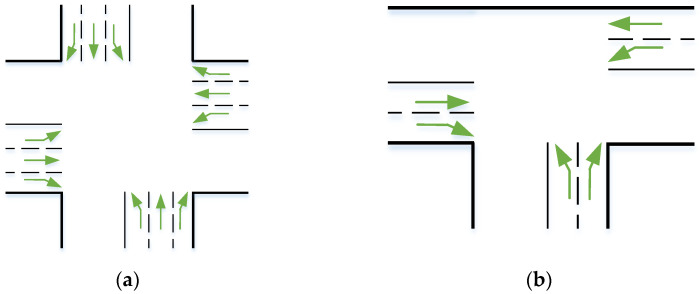
(**a**) A four-way junction with 8 roads and 12 possible traffic movements; (**b**) a three-way junction with 6 roads and 6 possible traffic movements.

The network of junctions is modelled as a directed graph of nodes $V=\left\{v_{1},v_{2},\ldots ,v_{N}\right\}$, in which each node represents a junction. The road starting from the junction $v_{i}$ to the adjacent junction $v_{j}$ is modelled as the link $r_{ij}=\left(v_{i},v_{j}\right)$. As demonstrated in Figure 1, each junction has certain traffic movements associated with it, and a subset of traffic movements that can occur simultaneously form a phase.

For driving habits, safety, and fairness, the composition of the phase scheme for a junction is usually fixed, and the sequence in the scheme cannot be adjusted frequently. The phase scheme of $v_{i}$ can be noted as $S_{i}=\left\{s_{ik}|k\in \left[1,\theta_{i}\right]\right\}$, where $\theta_{i}=\left|S_{i}\right|$, and the green time of the phase $s_{ik}$ at time t is noted as $g_{ik}\left(t\right)$, the value of which must be in $\left[g_{ik}^{min},g_{ik}^{max}\right]$. The task of RTSC is to reasonably optimize the green time of each phase between its maximum and minimum constraints according to the collected traffic flow parameters.

### 3.2. Phase-Based Sensing

**Figure 2:**
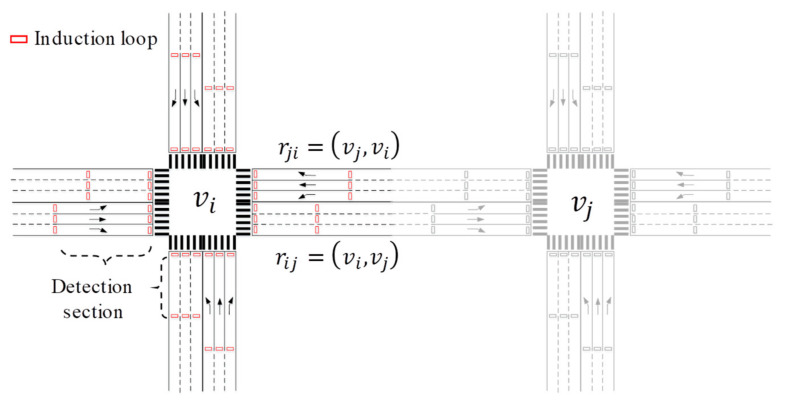
Traffic flow sensing based on induction loops.

Among traffic flow parameters, traffic volume is the most easily measured, and its detection error is relatively small. In our model, induction loops are placed at each entrance and exit of a junction to count the arrival and departure of vehicles in a section during each phase. As illustrated in Figure 2, the section is located behind the stop line or at the beginning of the exit. Thus, each road has two detection sections, which are called the start section and the end section, respectively.

The vehicle arrival rate, departure rate, and saturation of each section can be derived from traffic volume data easily. At time *t*, assume that the junction $v_{i}$ is running in its n-th signal cycle and that the duration of the phase $l(l\in S_{i})$ is $T^{i}(n,l)$. When the phase l is completed, the vehicle arrival rate of the *m*th lane at the start section of $r_{ij}$ can be calculated as:

(1) $$A_{ij}^{m}\left(n,l\right)=I_{ij}^{m}\left(n,l\right)/T^{i}(n,l)$$

where $I_{ij}^{m}(n,l)$ is the number of vehicles that drive through the junction and arrive at the *m*th lane at the start section of $r_{ij}$. Meanwhile, the vehicle departure rate of the *m*th lane at the end section of $r_{ji}$ can be calculated as:

(2) $$D_{ji}^{m}\left(n,l\right)=O_{ji}^{m}\left(n,l\right)/T^{i}(n,l)$$

where $O_{ji}^{m}(n,l)$ is the number of vehicles that depart from the *m*th lane at the end section of $r_{ji}$ and drive into the junction.

All data are collected and calculated independently based on phases, and there is no uniform sensing cycle for all junctions. In particular, detectors should also be installed on lanes that keep being released (such as right-turn lanes) so that the corresponding exit can estimate more accurately how many arriving vehicles are released in phases.

### 3.3. Phase Coordination Network

To coordinate phases within an area reasonably, it is critical to model their relationship first. Our approach is simple and intuitive—that is, we connect the signal phases of adjacent junctions into a network according to their influence on the same road. The following takes the road $r_{ij}$ in Figure 2 as example.

At time t, assume that the junctions $v_{i}$ and $v_{j}$ are running in their $\phi_{i}\left(t\right)$th and $\phi_{j}\left(t\right)$th cycles respectively. For the start section of $r_{ij}$, the average vehicle arrival rate of the most recent P cycles can be counted for the phase l of $v_{i}$:

(3) $$\alpha_{ij}\left(l,t\right)=\frac{1}{P}\sum_{p=1}^{P}\sum_{m=1}^{{\overset{⃐}{M}}_{ij}}A_{ij}^{m}\left(\phi_{i}\left(t\right)-p,l\right)$$

where $l\in S_{i}$ and ${\overset{⃐}{M}}_{ij}$ are the number of lanes at the start section of $r_{ij}$. Similarly, for the end section of $r_{ij}$, the average vehicle departure rate of $r_{ij}$ during the most recent P cycles can be counted for the phase l of $v_{j}$:

(4) $$\beta_{ij}\left(l,t\right)=\frac{1}{P}\sum_{p=1}^{P}\sum_{m=1}^{{\overset{⃑}{M}}_{ij}}D_{ij}^{m}\left(\phi_{j}\left(t\right)-p,l\right)$$

where $l\in S_{j}$ and ${\overset{⃑}{M}}_{ij}$ are the number of signal-controlled lanes at the end section of $r_{ij}$, and ${\overset{⃑}{M}}_{ij}$ may not equal ${\overset{⃐}{M}}_{ij}$.

For a road, its upstream arrival rate and downstream departure rate, which are affected by phases of upstream and downstream junction, will determine the number of vehicles stranded. Mismatched arrival and departure rates can easily cause road congestion. Therefore, the connection strength between the phases of $v_{i}$ and $v_{j}$ is defined as:

(5) $$w\left(s_{ik},s_{jr}\right)=\alpha_{ij}(s_{ik},t)\cdot \beta_{ij}\left(s_{jr},t\right)$$

where $k\in \left[1,\theta_{i}\right]$ and $r\in \left[1,\theta_{j}\right]$. The strength reflects the combined impact of two phases on the traffic flow of one road. The greater the strength, the greater the need for coordination between the two phases, as well as the potential utility after coordination, and vice versa. 

Repeat the above process for all roads, resulting in a network called the phase coordination network (PCN). The PCN is reconstructed periodically and does not have strong coupling constraints, such as uniform cycle and phase offset among connected phases.

## 4. Method

In the PCN, each directed link is associated with a corresponding actual road and its traffic sensing data, while each node is associated with a certain phase and its green time. Hence, the PCN actually provides a loosely coupled platform for RTSC by integrating all required input and output. Based on this platform, a two-stage RTSC method is proposed.

### 4.1. Phase Optimization Based on Complex Network

The load strategy is used to optimize the green time of phases. Firstly, we define the upstream and downstream loads of each link and node. Without loss of generality, the following takes the link $\left(s_{ik},s_{jr}\right)$ and node $s_{ik}$ as examples.

The upstream load of $\left(s_{ik},s_{jr}\right)$ is defined as the average vehicle saturation of the start section of $r_{ij}$ in the most recent P cycles:

(6) $$Lu\left({s_{ik},s}_{jr},t\right)=\frac{1}{P\theta_{i}}\sum_{p=1}^{P}\sum_{l=1}^{\theta_{i}}\sum_{m=1}^{{\overset{⃐}{M}}_{ij}}{\overset{⃐}{S}}_{ij}^{m}\left(\phi_{i}\left(t\right)-p,s_{il}\right)$$

where ${\overset{⃐}{S}}_{ij}^{m}(\phi_{i}\left(t\right),s_{il})$ is the saturation of the *m*th lane at the start section of $r_{ij}$ during the phase $s_{il}$ of the $\phi_{i}\left(t\right)$th cycle. Similarly, its downstream load can be defined as:

(7) $$Ld\left({s_{ik},s}_{jr},t\right)=\frac{1}{P\theta_{j}}\sum_{p=1}^{P}\sum_{l=1}^{\theta_{j}}\sum_{m=1}^{{\overset{⃑}{M}}_{ij}}{\overset{⃑}{S}}_{ij}^{m}\left(\phi_{j}\left(t\right)-p,s_{jl}\right)$$

On this basis, the local upstream load of $s_{ik}$ is defined as the sum of downstream load of all in-links:

(8) $${LU}^{1}\left(s_{ik},t\right)=\sum_{s\in {NU}^{1}\left(s_{ik}\right)}Ld\left(s,s_{ik},t\right)$$

where ${NU}^{1}\left(s_{ik}\right)$ is the set of upstream adjacent nodes of $s_{ik}$. By analogy, its local downstream load can be defined as:

(9) $${LD}^{1}\left(s_{ik},t\right)=\sum_{s\in {ND}^{1}\left(s_{ik}\right)}Lu\left(s_{ik},s,t\right)$$

where ${ND}^{1}\left(s_{ik}\right)$ is the set of downstream adjacent nodes of $s_{ik}$.

**Figure 3:**
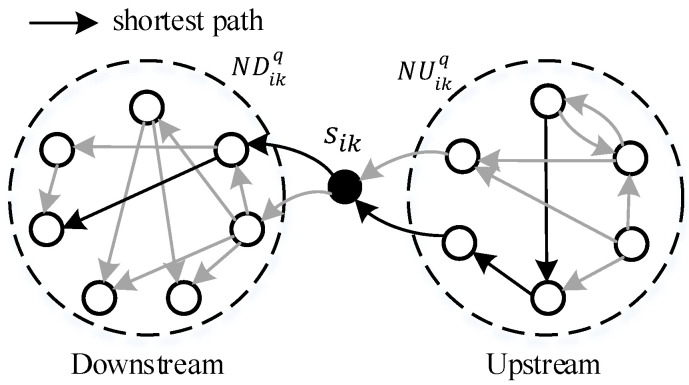
The upstream/downstream neighbor nodes of $s_{ik}$.

Then, the local load situation is extended to the neighbor network. Figure 3 illustrates the upstream and downstream neighbor nodes of $s_{ik}$. The *q*-order upstream neighbor nodes of $s_{ik}$ are the upstream nodes whose shortest directed distances do not exceed *q* steps, and they are noted as ${NU}^{q}\left(s_{ik}\right)$. Similarly, the *q*-order downstream neighbor nodes of $s_{ik}$ are noted as ${ND}^{q}\left(s_{ik}\right)$.

According to the shortest path, the local load of nodes can be generalized to the *q*-order neighbor network. The *q*-order upstream load of $s_{ik}$ is defined as:

(10) $${LU}^{q}\left(s_{ik},t\right)=\sum_{s\in {NU}^{q}\left(s_{ik}\right)\cup \left\{s_{ik}\right\}}f\left(s,s_{ik}\right){LU}^{1}\left(s,t\right)$$

where $f\left(s,s_{ik}\right)$ is the influence factor of s on $s_{ik}$ and is calculated as:

(11) $$f\left(s,s_{ik}\right)=\left\{\begin{array}{cc} \prod_{r\in R\left(s,s_{ik}\right)}w\left(r\right) & s_{ik}\neq s\\ 1 & s_{ik}=s \end{array}\right.$$

where $R\left(s,s_{ik}\right)$ is the shortest directed path from s to $s_{ik}$. Similarly, the *q*-order downstream load of $s_{ik}$ can be defined as:

(12) $${LD}^{q}\left(s_{ik},t\right)=\sum_{s\in {ND}^{q}\left(s_{ik}\right)\cup \left\{s_{ik}\right\}}f\left(s_{ik},s\right){LD}^{1}\left(s,t\right)$$

The above calculation process depends on the topology of the PCN. Algorithm 1 gives a breadth-first search method to compute the *q*-order downstream load of $s_{ik}$, and the computing of *q*-order upstream load is similar.

Finally, the load strategy is used to optimize the green time of $s_{ik}$ through the S-shaped function:

(13) $$g_{ik}\left(t\right)=g_{ik}^{min}+\frac{g_{ik}^{max}-g_{ik}^{min}}{1+e^{{LD}_{ik}^{q_{1}}\left(t\right)-{LU}_{ik}^{q_{2}}\left(t\right)}}$$

where $q_{1}$ and $q_{2}$ are positive integers. If $q_{1}=q_{2}$, it is a load balancing strategy. In this case, when the upstream load exceeds the downstream load, the green time is near $g_{ik}^{max}$. Otherwise, it is close to $g_{ik}^{min}$. If $q_{1}<q_{2}$, the load balancing strategy will become the upstream priority strategy, and vice versa.
**Algorithm 1.** Compute the *q*-order downstream load of $s_{ik}$for each node s in PCN s.color ← WHITE; s.depth ← 0; s.pre ← NIL; $s_{ik}$.color ← GREEN; Q←∅; ND←∅; ENQUEUE(Q,$s_{ik}$); while Q≠∅ u = DEQUEUE(Q); if u.depth ≥
*q*  continue; for each node v in ${ND}^{1}\left(u\right)$  if v.color = WHITE   v.color ← GREEN; v.depth ←u.depth + 1; v.pre ←u;   ENQUEUE(Q,v);  ENQUEUE(ND,u); while ND≠∅ u = DEQUEUE(ND); compute $f\left(s_{ik},u\right)$ by backtracking u.pre till $s_{ik}$; ${LD}^{q}\left(s_{ik},t\right)\leftarrow {LD}^{q}\left(s_{ik},t\right)$ + ${f\left(s_{ik},u\right)\cdot LD}^{1}\left(s_{ik},t\right)$; return ${LD}^{q}\left(s_{ik},t\right)$;

### 4.2. Phase Execution Based on Fuzzy Control

The phase optimization is carried out immediately after the construction of the PCN, with a period of approximately 5–15 min. However, the control cycle of a single junction is usually less than 150–240 s, meaning that the interval between the global phase optimizations above is relatively long. If the optimization result is directly used in the signal control of a single junction, it will inevitably lead to a certain lag and error, and it is difficult for signal control to follow the rapid changes in the traffic situation at the junction. To improve real-time performance, the optimized green time is used as a guide and combined with the fuzzy control method to achieve fine-grained phase execution.

**Figure 4:**
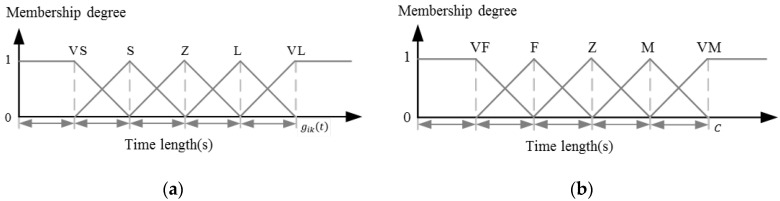
(**a**) The fuzzy membership function of the length of green time; (**b**) the fuzzy membership function of the vehicle numbers in a detection section.

Firstly, the length of green time of a phase is divided into five fuzzy language values: *VS* (very short), *S* (short), *Z* (middle), *L* (long), and *VL* (very long). Their membership functions, taking the phase $s_{ik}$ as an example, are shown in Figure 4a, where the guide $g_{ik}\left(t\right)$ is used as the inflection point value for *VL*. In addition, the number of vehicles in the detection section of each lane is also divided into five fuzzy language values: *VF* (very few), *F*(few), *Z*(middle), *M*(many), and *VM* (very many). A membership function similar to that shown in Figure 4b has been used, in which the maximum number of vehicles that can be held in the detection section C is used as the inflection point value for *VM*.

At the beginning of a phase, the number of vehicles currently released on the lanes and those waiting to be released can be obtained by the detectors of end sections, denoted as GN and RN, respectively. Then, following the fuzzy control rules in Table 2, the final green time for a phase to be executed can be determined. Both the fuzzification and defuzzification processes employ the method of maximum membership degree.

Our method differs from the existing research on applying fuzzy control to traffic signal control, which often decides whether to extend the green light or not in a short time before the end of phases. In our method, the green time is decided directly at the beginning of phases, so it does not affect the use of countdown displays in practice, which must know the exact green time in advance (usually no less than 10 s). Moreover, benefiting from the optimized green time, the fuzzy rule table can adopt a simple greedy strategy, i.e., when GN≥RN, it can be directly set to the green light time at the same level. There is no need to customize fuzzy rules and membership functions based on expertise for different junctions.

### 4.3. Flexibility and Optimality

Integrating the aforementioned two methods, taking the junction $v_{i}\in V$ as an example, the proposed traffic signal coordination control is shown in Algorithm 2.
**Algorithm 2.** Traffic signal coordination control of the junction $v_{i}$$S_{i}=\left\{s_{ik}|k\in \left[1,\theta_{i}\right]\right\}$ is the phase scheme of $v_{i}$ $\theta_{i}=\left|S_{i}\right|$ while *True* for *k*$=1:\theta_{i}$ According to Algorithm 1, calculate the $q_{1}$-order downstream load of $s_{ik}$; Similar to Algorithm 1, calculate the $q_{2}$-order upstream load of $s_{ik}$; Calculate the optimized green light time $g_{ik}\left(t\right)$ of $s_{ik}$ according to Formula (13); Get the GN and RN by detectors of end sections; Input $g_{ik}\left(t\right)$, GN, and RN into the fuzzy controller to determine the final green light time of $s_{ik}$; The traffic signal controller sequentially outputs green, yellow, and red lights for the corresponding time period.; Update vehicle arrival rate, departure rate, and saturation for each lane during $s_{ik}$ for iterative  updating of PCN.

In research, we have always kept the principles of simplicity and practicality in mind. In the PCN model, the traffic volume data used are a type of traffic flow parameter that is easy to collect and that has high accuracy. In addition to induction loops, many vehicle detectors, such as video detectors, ultrasonic detectors, radar detectors, etc., can detect traffic volume. When constructing the PCN, for roads that cannot be sensed, all detection data can simply be set to zero. Similarly, for junctions that cannot be included in the RTSC, it can be assumed that only an empty phase is contained.

In the RTSC method based on the PCN, we further keep the signal control at each junction loosely coupled for flexibility. This approach ensures that the entire method is highly adaptable to the engineering and application complexity that may arise in actual transportation infrastructure.

The designed RTSC method also follows the simplicity and practicality principles. Specifically, we employ load strategies for global phase optimization and fuzzy rules for local phase execution. These straightforward methods are highly consistent with the experience of transportation managers and are expected to yield promising optimization results. In Section 6, we will verify it through numerous computational experiments.

## 5. Prototype

The transportation control infrastructure of a city is constantly evolving due to various reasons, such as the installation of new detectors or controllers on new roads or junctions or the removal or upgrading of existing ones. To ensure smooth functioning of the RTSC system, it must adapt accordingly to these changes in infrastructure. Therefore, the maintainability of the RTSC system is also very critical in actual long-term operation.

### 5.1. Architecture

**Figure 5:**
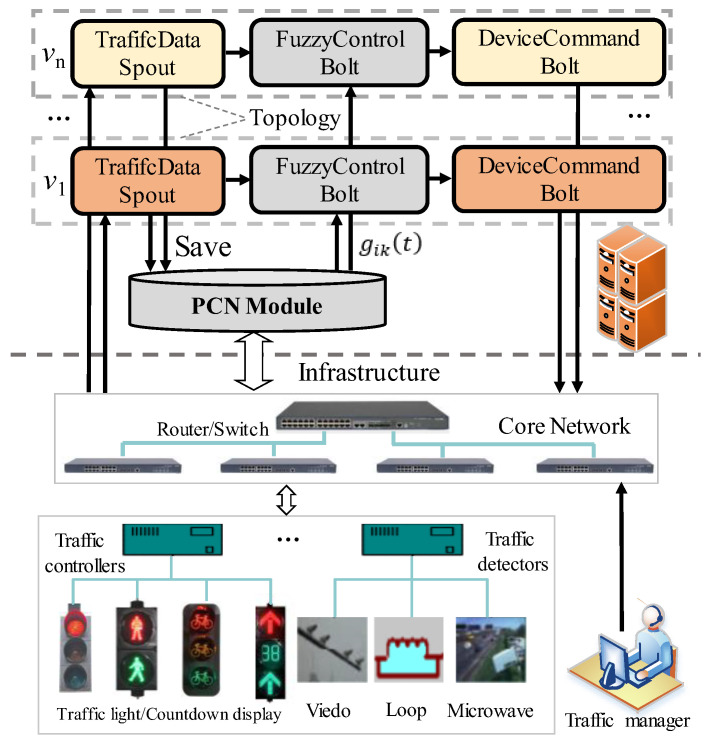
The architecture of the RTSC system.

The flexible stream-computing framework, called Storm [42], is introduced to develop the RTSC system, the architecture of which is presented in Figure 5.

The signal control of each junction corresponds to a topology composed of three types of nodes. Firstly, the *TrafficDataSpout* node is responsible for receiving and processing all traffic detection data at the corresponding junction. It then passes the (GN,RN) to the *FuzzyControlBolt* node, which decides the green time of the current phase according to the fuzzy rules and the optimized green time. Finally, the green time for execution is passed to the *DeviceCommondBolt* node, which is responsible for translating the results into instructions for the corresponding controller and issuing them through the network. In addition to this, the PCN module is responsible for storing historical traffic detection data and periodically updating the phase coordination network to optimize the green time of phases. This ensures that the system can adapt to changes in traffic flow and adjust the signal control timing accordingly. Overall, this architecture provides a flexible and efficient approach to managing traffic flow in a transportation control infrastructure.

The RTSC system can be customized to accommodate different equipment manufacturers by customizing the nodes, such as the *TrafficDataSpout* and *DeviceCommandBolt* nodes. The steam computing architecture allows for dynamic addition or removal of junctions, making it easy to expand or modify the network. Additionally, each node has a backup mechanism that can automatically restart in case of failure, providing high fault tolerance for the entire system. These features make the RTSC system highly adaptable and reliable, ensuring smooth operation in various transportation control environments.

### 5.2. Time Complexity

To analyze the time complexity of the proposed RTSC method, we consider an extreme case in which all traffic detectors collect and upload data directly to the system without any processing and in which all traffic signal controllers only request and receive green time information from the RTSC system without making any local decisions. In this case, the RTSC system has the largest computational load.

The workflow of the RTSC system is event-driven and distributed in clusters. When the *TrafficDataSpout* receives traffic detection data, it calculates the volume and saturation of vehicles in each start and end section. The time complexity is $O\left(M\right)$, where M is the total number of sections. When a request is received from a traffic signal controller, the message is forwarded to the corresponding topology for processing, and the time complexity is $O\left(N\right)$, where N is the whole number of fuzzy language values in fuzzy control. When the PCN module needs to be updated, the calculation includes the construction of the PCN and the optimization of the green time of each phase. The former is calculated serially, and the time complexity is $O\left(K_{1}\Omega \right)$, where Ω is the total number of phases in the area and $K_{1}\left(K_{1}\ll \Omega \right)$ is the average number of the 1-order downstream neighbor nodes of each node. The latter is calculated in parallel, and the time complexity of each parallel unit is $O\left(K_{q}PM\theta \right)$, where $K_{q}\left(K_{q}<\Omega \right)$ is the average number of *q*-order upstream and downstream nodes of each node and θ is the average number of phases in a signal cycle.

Based on the analysis, it can be concluded that the entire RTSC method has a low time complexity of no more than $O\left(\Omega^{2}\right)$, resulting in better real-time performance. In practice, traffic detectors and signal controllers often have certain edge-computing capabilities and can further share the computing load, further reducing the time complexity of the system.

## 6. Computational Experiments

For legal and safety reasons, it is almost impossible to long-term test an RTSC system in practice, so it is common to use traffic simulation software. TransWorld, a microscopic traffic simulation software based on artificial transportation system theory [43]. This software is thus used to validate the RTSC system in terms of adaptability and performance. As an experimental platform, TransWorld has been put into use in many major projects, such as urban traffic control, large-scale sports events, and plays an important role in the simulation, analysis, and prediction of traffic demands [44,45]. TransWorld provides a traffic flow detection interface and a traffic signal switching interface based on network APIs. With these two interfaces, the developed RTSC system can conduct real-time traffic signal control experiments on the traffic scenes simulated byTransWorld. After the experiment, TransWorld also provided detailed traffic statistical indicators for result analysis.

### 6.1. Experiment Setting

**Figure 6:**
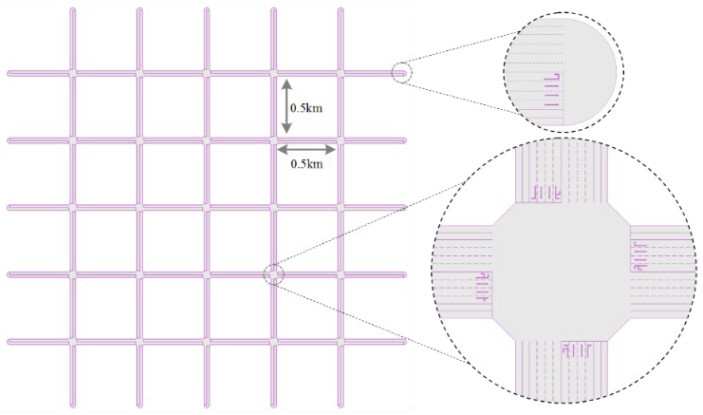
The road network used for simulations.

As illustrated in Figure 6, a 5×5 grid road network is built with 25 junctions and 20 boundary nodes in total. The distance between any two adjacent nodes is 0.5 km, and the whole area is 9 km^2^, which is a relatively dense road network. There are four channelized lanes at the entrances of each junction. The lengths of each start and end section are set to 60 m and 120 m, respectively, and the maximum number of vehicles in each section is converted according to an average of 6 m per vehicle. Uniformly, the minimum and maximum green times for all phases are set to 15 s and 45 s, respectively, and their yellow and red times are both set to 3 s. The sliding time window in PCN is set to 3 periods, i.e., P=3.

**Figure 7:**
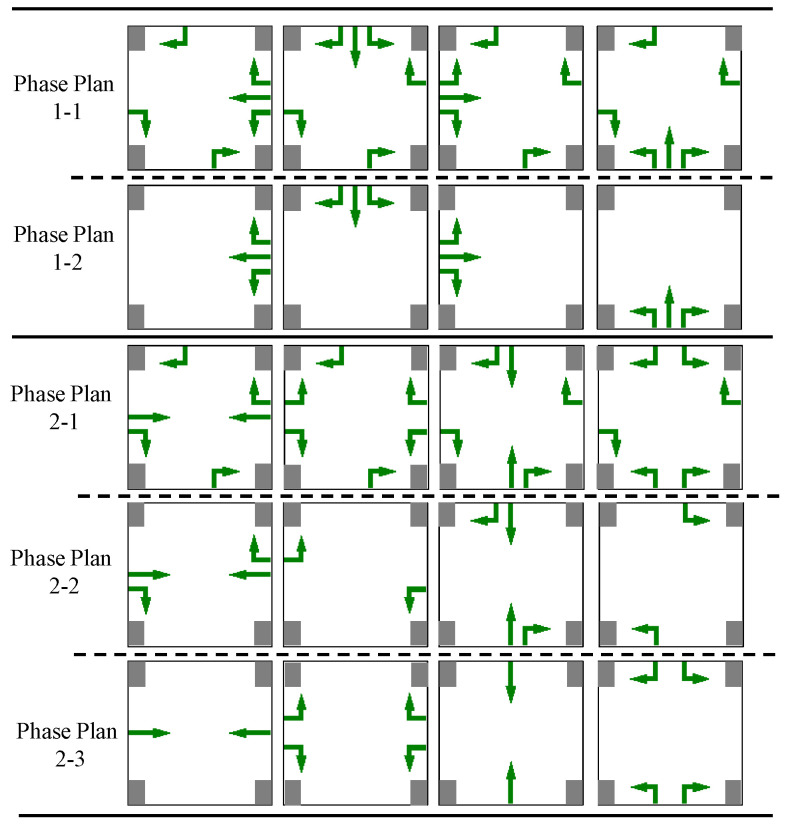
Five typical phase schemes for an intersection.

In addition, computational experiments are configured following the triple random principle:

1. Randomly generate vehicles crossing boundary nodes. Vehicles are randomly generated at each boundary node according to the Poisson distribution, and other boundary nodes are randomly selected as destinations. The entire area is set to generate an average of 20×700 vehicles per hour, and the boundary node with the largest total generation is no more than twice the minimum boundary node. The simulation duration is 22 h.
2. Randomly select phase schemes for junctions. There are two typical four-phase configurations for a four-way junction, i.e., release in opposite directions at the same time and release each direction in turn. Depending on whether the right turn is signal-controlled or not, a total of five configurations can be derived, as shown in Figure 7. The 25 junctions randomly select one of the five phase schemes as their own scheme.
3. Randomly select some junctions or roads, assuming that there are special circumstances of engineering or application complexity. The associated junctions will be excluded from the RTSC, and their signal control could be degraded to traditional traffic signal full-actuated control, since the same traffic detectors as full-actuated control are used. The minimum and maximum green times of each phase remain unchanged, and the unit extension of green time is set to 3 s.

### 6.2. Adaptability Analysis

**Figure 8:**
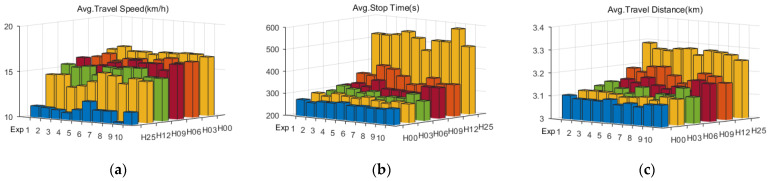
(**a**) Average travel speed of Exp1–10; (**b**) average stop time of Exp1–10; (**c**) average travel distance of Exp1–10. The colors are used to indicate different subgroups, namely H00–H25.

To verify the adaptability, 10 groups of experiments (denoted as Exp1–10) are designed with random traffic flow distributions and phase configurations. Each group experiment is further divided into 6 subgroups, randomly assuming that there are 0%, 3%, 6%, 9%, 12%, and 25% junctions that are excluded from the RTSC, denoted as H00, H03, H06, H09, H12, and H25, respectively. To reduce random errors, each experiment is repeated 10 times, resulting in a total of 600 (10 × 6 × 10) experiments. At the end of every 10 experiments, the average travel distance, speed, and stop time of all passing vehicles are calculated and drawn in Figure 8.

In terms of average travel speed and stop time, with the increase in complexity (H00, H03, H06, H09, H12), the average travel speed decreases gradually and the average stopping time increases gradually. However, all of them are better than H25. TransWorld simulates situations in which people are forced to change lanes due to long queues or are unable to change lanes due to congestion, which can result in differences in the average travel distances of vehicles within the same subgroup of experiments. In 10 groups of experiments, the average travel distances of H00–H12 are all less than that of H25, indicating that the proposed RTSC method can alleviate regional traffic congestion in time and reduce times to change paths for traffic congestion.

**Figure 9:**
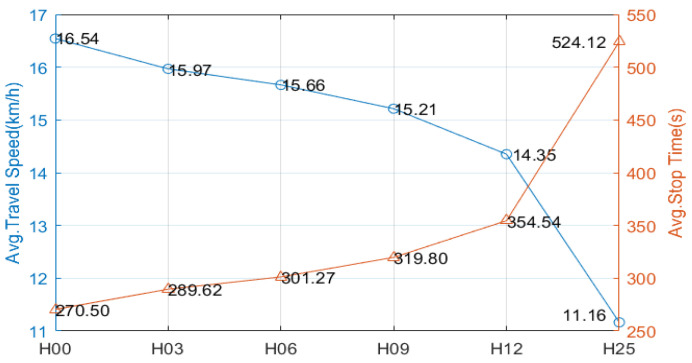
Comparison of experimental results according to complexity.

Ignoring traffic flow distribution and phase configuration, a statistical comparison can be made based solely on the degree of complexity. As shown in Figure 9, when the complexity increases from 0% (H00) to 48% (H12), the average travel speed decreases from 16.54 km/h to 14.35 km/h (decreased by 13.2%) and the average stop time increases from 270.50 s to 354.54 s (increased by 31.1%). However, this is still significantly better than H25 (100%).

**Figure 10:**
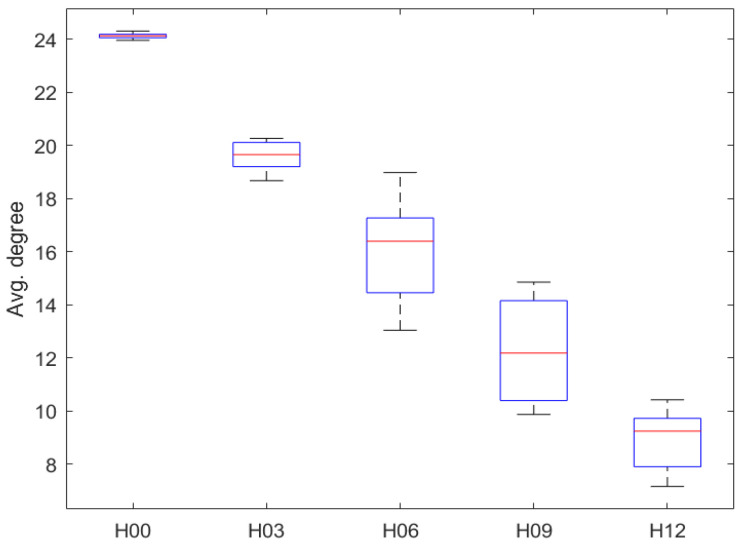
The average degree of the PCN according to complexity.

The average degree of a network is a useful metric that measures the number of edges compared to the number of vertices. This can provide insight into the connectivity density between nodes within the network. In Figure 10, we observe how the average degree changes as the size of the network increases.

As the complexity of the transportation control infrastructure increases, the average degree of the PCN gradually decreases. However, each phase node in H12 is still connected to about nine phase nodes on average and has the basis for signal coordination. In conclusion, the proposed RTSC method exhibits strong adaptability and can effectively optimize traffic signal control for varying degrees of complexity in transportation control infrastructure.

### 6.3. Performance Analysis

In addition to adaptability, the performance of the RTSC in improving traffic efficiency through coordinated control is also very critical. In the previous test, we only adopted the load-balancing strategy ($q_{1}=3,\;q_{2}=3$). To test the performance more comprehensively, we use the same process to test the other three control strategies: (1) an upstream priority strategy ($q_{1}=1,\;q_{2}=3$); (2) a downstream priority strategy ($q_{1}=3,\;q_{2}=1$); (3) and a no-coordination strategy, in which the result of phase optimization is fixed to the respective maximum green time.

Another 10 groups of experiments (denoted as Exp11–20) are designed with random traffic flow distributions and phase configurations, and each group experiment is further divided into four subgroups. The four subgroups respectively run four types of strategies. Similarly, each experiment is repeated 10 times to reduce random errors, and a total of 400 (10×4×10) experiments are carried out. In these experiments, all junctions are included in the RTSC.

**Figure 11:**
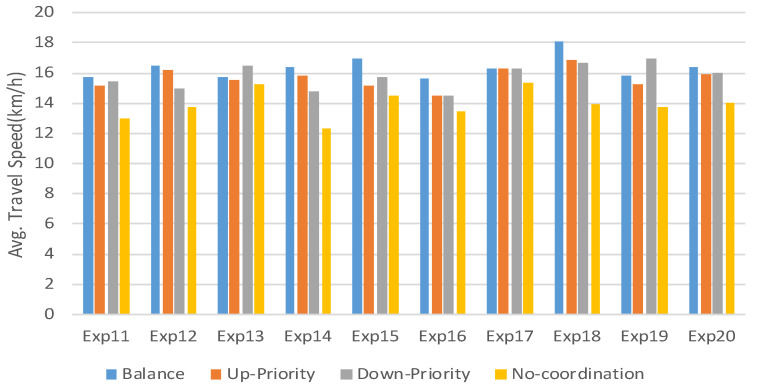
Comparison of 4 strategies.

At the end of every 10 experiments, the average travel speed can be obtained and a statistical comparison can be made, as shown in Figure 11. It can be found that under different traffic flow distributions and phase configurations, no strategy can always stay ahead. This is consistent with the experience that there is no one-size-fits-all strategy in actual traffic management. However, it is worth pointing out that among the three load strategies, the load-balancing strategy has never been the last one. Moreover, the three load strategies outperform the no-coordination strategy, indicating that the designed coordination mechanism has played an important role.

### 6.4. Discussion

In the experiments, we did not perform tests in which more than 50% of junctions could not be connected to the RTSC system. If this happens in practice, it means that the entire transportation system has encountered a very special situation that exceeds the scope of the RTSC, and H12 (48%) is sufficient to verify whether the proposed method has adequate flexibility to ensure universal applicability.

We do not simulate the process of complexity that occurs in the transportation control infrastructure but exclude the associated junctions from the RTSC directly. This unified processing method is simple, but it also loses some useful traffic information. For example, when a junction enters the manual control mode, its associated detectors still normally collect traffic flow parameters.

Given that the generated traffic flow distribution is uniform and random throughout the simulation period, the junctions that are excluded from the RTSC are confirmed at the beginning of the simulation rather than appearing randomly during the simulation. This can avoid assuming the start time and duration of the exception. Otherwise, it will lead to a combination explosion (since the time is continuous), and it is not helpful to obtain a statistically significant conclusion.

## 7. Conclusions

The paper proposes a flexible RTSC method based on the innovative phase coordination network for achieving effective traffic signal coordinated control even with complicated transportation control infrastructure. The proposed model and method are both built around phases, beginning with modelling the relationship between phases based on data, then using load strategies to optimize the green time of phases, and finally applying fuzzy rules to optimize the execution of phases. Data, heuristic strategies, and rules drive the entire process. Furthermore, using stream-computing technology, an easy-to-maintain RTSC system is developed.

From method to system, practicality has always been the top priority. At present, the application of complex network theory in our method is still relatively preliminary. Though it is consistent with intuition and practical experience, it still lacks rigorous theoretical derivation and proof, which is one of the next steps to be carried out based on the theory of complex network dynamics. On the other hand, the academic community does not yet have a unified testing platform to compare different RTSC methods fairly. This is a very meaningful research direction that needs to be filled, and we are very interested in doing some work in this area based on the existing TransWorld in the future.

## 8. Patents

A Regional Traffic Signal Coordination Control Method and System for Complex Traffic Control Environments, China: ZL 202110097004.6, 22 August 2022.

## Figures and Tables

**Table 1:** RTSC Systems in The World.

Nation	RTSC Systems	Nation	RTSC Systems
**UK**	TRANSYT, SCOOT	**USA**	RT-TRACS, REHODS, OPAC
**Japan**	STEAM, KATNET	**Germany**	MOTION
**Canada**	RTOP	**Italy**	SPOT/UTOPIA
**Australia**	SCATS	**Spain**	ITACA, SANCO
**France**	PRODYN	**China**	NUTCS, SMOOTH, HiCon

**Table 2:** Fuzzy control rule table based on greedy strategy.

GN	RN				
	VF	F	Z	M	VM
VF	VS	VS	VS	VS	VS
F	S	S	VS	VS	VS
Z	M	M	M	S	S
M	L	L	L	L	M
VM	VL	VL	VL	VL	VL

## Data Availability

The data are available from the corresponding author on reasonable request.
